# Delivery of Personalized Care for Locally Advanced Rectal Cancer: Incorporating Pathological, Molecular Genetic, and Immunological Biomarkers Into the Multimodal Paradigm

**DOI:** 10.3389/fonc.2020.01369

**Published:** 2020-08-14

**Authors:** Éanna J. Ryan, Ben Creavin, Kieran Sheahan

**Affiliations:** ^1^School of Medicine, University College Dublin, Dublin, Ireland; ^2^Department of Surgery, Royal College of Surgeons in Ireland, Dublin, Ireland

**Keywords:** chemotherapy, radiation, rectal cancer, mesorectal excision, prognostic markers, personalized medicine, survival

## Abstract

Approximately one-third of all newly diagnosed colorectal cancer (CRC) is composed of rectal cancer, with the incidence rising in younger patients. The principal neoadjuvant treatments consist of neoadjuvant short-course radiotherapy and long-course chemoradiation. Locally advanced rectal cancer (LARC) is particularly challenging to manage given the anatomical constrictions of the pelvis and the risk for local recurrence. In appropriately treated patients, 5- and 10-year overall survival is estimated at 60 and 50%, respectively. The prognosis for LARC has improved in recent years with more access to screening, advances in surgical techniques, and perioperative care. Furthermore, the refinement of the multidisciplinary team with combined-modality management strategies has improved outcomes. These advancements have been augmented by significant improvements in the understanding of the underlying tumor biology. However, there are many instances where patient outcomes do not match those for their tumor stage and accurate prognostic information for individual patients can be difficult to estimate owing to the heterogeneous nature of LARC. Many new combinations of chemotherapy with radiotherapy, including total neoadjuvant therapy with targeted therapies that aim to diminish toxicity and increase survival, are being evaluated in clinical trials. Despite these advances, local recurrence and distant metastasis remain an issue, with one-third of LARC patients dying within 5 years of initial treatment. Although much of the new pathological, molecular genetics, and immunological biomarkers allow refinement in the classification and prognostication of CRC, the relative importance of each of these factors with regards to the development and progression of LARC remains incompletely understood. These factors are often insufficiently validated and seldom consider the individual characteristics of the host, the tumor and its location, the local available expertise, or the probable location of recurrence. Appreciating the mechanisms behind these differences will allow for a more comprehensive, personalized approach and more informed treatment options, leading to ultimately superior outcomes. This review aims to first outline the current multidisciplinary context in which LARC care should be delivered and then discuss how some key prognosticators, including novel histopathological, molecular genetics, and immunological biomarkers, might fit into the wider context of personalized LARC management in the coming years.

## Introduction

Colorectal cancer (CRC) is the second highest cause of cancer-related mortality in Europe with an estimated 500,000 cases in 2018 (1). Approximately one-third of all newly diagnosed CRC is composed of rectal cancer with the incidence rising in younger patients throughout the western world (2). The principal neoadjuvant treatments consist of neoadjuvant short-course radiotherapy and long-course chemoradiation (nCRT) (3–6). The latter is mainly used in the treatment of locally advanced rectal cancer (LARC), defined for the purpose of this review as clinical stage T3–4 or any clinical T stage with node-positive disease (≥cT_3−4_ or any cT with cN_1/2_). LARC is particularly challenging to manage given the anatomical constrictions of the pelvis and the risk for local recurrence. Neoadjuvant therapy followed by total mesorectal excision (TME) with either low anterior resection (LAR) or abdominoperineal excision is associated with improved survival (3–6). In appropriately treated patients, 5- and 10-year overall survival (OS) is estimated at 60 and 50%, respectively (5). Aside from this survival benefit, nCRT may reduce local recurrence (LR) rates, downsize the tumor, and facilitate subsequent successful R0 resection with sphincter preservation (7).

The prognosis for LARC has improved in recent years with more access to screening, advances in surgical technique and perioperative care, along with the refinement of the multidisciplinary team with combined-modality management strategies. These advances have been augmented by significant improvements in the understanding of the underlying tumor biology reflected by new pathological, molecular genetics, and immunological biomarkers (8, 9). However, there are many patients whose outcomes do not match those typical for their tumor stage and accurate prognostic information for individual patients can be difficult to estimate owing to the heterogeneous nature of the disease. Many new combinations of chemotherapy with radiotherapy, including total neoadjuvant therapy (TNT) with targeted therapies that aim to diminish toxicity and increase survival, are being evaluated in clinical trials (10, 11). Despite these advances, local recurrence and distant metastasis remain an issue, with one-third of LARC patients dying within 5 years of initial treatment (10).

Although much of the new molecular and immunological data allows refinement in the classification and prognostication of CRC, the relative importance of each of these factors with regards to the development and progression of LARC remains incompletely understood. Current excitement about novel prognostic markers, such as tumor budding, The Cancer Genome Atlas' Consensus Molecular Subtypes (CMS), and the Oncotype DX (Genomic Health, Redwood, California, USA), and Immunoscore tests (Integrative Cancer Immunology Laboratory, INSERM, Paris, France) underestimate the complexity of the disease (8). These factors are often insufficiently validated and seldom consider the individual characteristics of the host, the tumor and its location, the local available expertise, or the probable location of recurrence (9). Appreciating the mechanisms behind these differences will allow for a more comprehensive, personalized approach and more informed treatment options, leading to ultimately superior outcomes.

This review aims to first outline the current multidisciplinary context in which rectal cancer care should be delivered and then discuss how some key prognosticators of LARC, including novel histopathological, molecular genetics, and immunological biomarkers, might fit into the wider context of personalized LARC management in the coming years.

## Multidisciplinary Assessment, Histopathology, and Staging

### The Multidisciplinary Team

A multidisciplinary approach to rectal cancer is crucial owing to the complexity of the disease. Improved radiological staging, mesorectal grading, and oncological outcomes can be mainly attributed to the development of the multidisciplinary approach to LARC management, incorporating surgeons, pathologists, radiologists, and oncologists. Therefore, a multimodality approach is crucial in providing personalized and effective treatment to rectal cancer patients (12).

### Radiological Staging and Assessment

MRI is the preferred imaging modality for pelvic staging in LARC before treatment, aiding in deciding the need for neoadjuvant therapy while also potentially predicting patients who have worse outcomes. Good response to neoadjuvant therapy is seen in node negative patients with more superficially located tumors. Free resection margins (CRM) and the absence of adverse pathological features on MRI are all predictors of good response to treatment (13, 14). The combination of extramural venous invasion (EMVI), involvement of regional lymph nodes, and higher T stages on MRI are associated with synchronous metastatic disease and worse outcomes (15). MRI maintains a high specificity and moderate sensitivity for the detection of EMVI (16), and MRI-detected EMVI (mrEMVI) in particular may represent an independent adverse feature whose prognostic and predictive importance (to nCRT response) is somewhat underestimated (14, 15). MRI, however, is limited when restaging patients after neoadjuvant therapy owing to its failure to accurately differentiate residual tumor from post–nCRT-related desmoplastic reactions, inflammation, and fibrosis (17). The accuracy of correctly staging the tumor and nodes is roughly 45–67% and 76–93% when standard MRI modalities are used (18). However, for CRM, mean sensitivity and mean specificity in a meta-analysis of over 1,500 patients were 76.3 and 85.9%, respectively (19). Furthermore, sensitivities of 21–55% and specificities of 76–93% are reported when MRI is used to accurately identify response to neoadjuvant therapy (18, 20). Interestingly, the diagnostic performance of MRI for mrEMVI evaluated after the nCRT is good and may have additional prognostic impact (21–23). Diffusion weighting is one newer imaging method that has been shown to improve re-staging accuracy overall (19).

### Histopathology and Staging

The recent fifth edition of the WHO Classification of Digestive System Tumours reflects important recent advancements in our understanding of digestive system cancers. For the first time, certain digestive tumor types including CRC are defined as much by their molecular phenotype as their histological characteristics (24). However, histopathologic assessment remains the “gold standard” for diagnosis of LARC while the extent of the disease anatomically, as assessed by clinicopathological staging, remains the most useful prognosticator. Macroscopic evaluation of the resected specimen is still an extremely important aspect in histopathological staging. Both the grade and distance from the circumferential resection margin (CRM) impact survival, LR, and adjuvant therapy decisions. An intact mesorectum is the gold standard as it has been shown to reduce local recurrences to <10% (25). Therefore, its correct evaluation has huge implications for patients, especially in the era of minimally invasive surgery (26). A clear CRM is associated with significantly improved local recurrences and oncological outcomes, although consensus on the distance from this margin is still controversial (27).

### Tumor Budding

Tumor budding is a histopathological feature of epithelial cancers where tumor cells or cell clusters of less than five cells detach from the invasive margin and migrate into the peritumoral stroma (28–30). It is thought to correspond to the initial phase of tumor invasion and has been reported to be relevant to metastatic activity and prognostic outcome in CRC (28–30). The process is hypothesized to involve the epithelial–mesenchymal transition resulting in resistance to apoptosis, potentially impacting negatively on radiotherapy response (29). The widespread implementation of tumor budding as an adjunct to the TNM classification has been hindered by a lack of consensus definition, reproducibility, and method of assessing budding. Recent consensus recommendations for assessing budding include protocols for incorporating tumor budding into CRC pathological reporting (31). The evaluation of tumor budding in pre-treatment biopsies is also possible and may help identify rectal cancer patients with worse outcomes or who will not respond to therapy, thus providing the opportunity to deliver individualized care for these patients (29).

### Lymphovascular, Venous, and Perineural Invasion

Lymphovascular invasion (LVI) is defined as tumor cell invasion into the lymphatic and/or blood vessels. It is regarded as an adverse pathological finding and has a crucial part in the development of metastatic disease (32). The presence of LVI is significantly associated with worse OS and disease-free survival (DFS) (33). EMVI is defined pathologically as tumor cells present in the vasculature outside the muscularis propria. When present, it produces more locally advanced tumors that invade the mesorectum, ultimately impacting negatively on survival and recurrence rates (34). Pathological assessment of EMVI has resulted in under reporting of cases mainly caused by a lack of pathological definition and staining methods used (35). Perineural invasion (PNI) is defined as the neoplastic invasion of nerves or nerve sheaths. Although it is a significant pathological finding, it is under reported in the majority of cases (0.05%) (36). PNI is a key pathological feature in a number of cancers outside of CRC. The presence of PNI represents a process for neoplastic invasion and spread outside the traditional lymph and blood vessel route. Worse OS and DFS is encountered in patients with PNI (37).

### Tumor Deposits

Tumor deposits were first described in 1935, although their characterization and clinical significance have changed over the years. Tumor deposits are defined as isolated tumor foci found in the pericolic or perirectal fat or in the mesocolic fat/adjacent mesentery away from the invasive margin of the tumor without evidence of residual lymphatic tissue (38). In the absence of ≥1 positive lymph node, they are documented as N1c (i.e., not in T stage category, but rather in the higher N stage category) (39). The presence of tumor deposits is associated with a higher tumor stage, metastatic disease, and a poorer prognosis (40).

## Multimodal Therapy, Pathologic Complete Response, and Organ Preservation

The standard multimodality therapy for LARC generally includes nCRT (usually 50–54 Gy and 5-fluorouracil) followed by surgery in the TME plane with or without adjuvant chemotherapy (5, 6). With this treatment paradigm, the incidence of local recurrence is <10% and, in approximately 15% of patients, a pathologic complete response (pCR) may be observed (3–6). In patients achieving a pCR, improved LR rates have been reported. Consequently, pCR has become a well-established surrogate for DFS and interest in achieving this outcome has grown (41).

It is now understood that tumor regression is time dependent and a longer waiting time between nCRT and surgery has been one method proposed to increase rates of pCR (42, 43). Intensification of the chemotherapy regimen has also been suggested (44). Of the six large phase-III RCTs comparing nCRT with or without oxaliplatin, only the CAO/ARO/AIO-04 study reported an improved survival with additional chemotherapy and no phase-III RCT of concurrent irinotecan has been reported (10). Consequently, single-agent 5-fluouracil/capecitabine-based nCRT remains the standard of care in LARC. A new variation of this approach aimed at reducing the instance of micrometastatic disease and local recurrence rates is TNT. This consists of chemotherapy administered either before (induction chemotherapy) or after (consolidation chemotherapy) nCRT and before surgery (45). Weighted mean local recurrence and distant failure rates of 3.5 and 20.6%, respectively, have been reported (11). Consequently, this new strategy is increasingly being considered in patients with high-risk LARC owing to improved compliance with chemotherapy and disease control.

In patients with a clinical complete response (cCR) to nCRT on endoscopic and radiological examination, organ preservation, via local excision or active surveillance, has emerged in an effort to avoid the morbidity and quality of life issues associated with TME (46, 47). The long-term outcomes of a large, international, multi-institutional expectant “watch and wait” database with more than a thousand patients recently demonstrated that LR occurs mostly in the first 2 years, primarily in the bowel wall, underscoring the necessity of endoluminal examination to detect local regrowth early and at a salvageable stage. Local unsalvageable disease after this strategy was infrequent (12%), with a 5-year OS of 85% (47).

However, the majority of patients have less favorable responses to treatment, and local recurrence and distant metastasis remain an issue. Some patients may benefit from “straight up” surgery or alternate neoadjuvant and adjuvant therapies to increase response rates and survival, as well as to avoid the morbidity associated with standard treatment (48–50). For these reasons, personalizing chemotherapy regimens with surgery or organ preservation likely represents the future of rectal cancer management. Molecular, genetic, and immunological biomarkers are required to help guide patient selection for these novel therapeutic strategies in an efficacious and cost-effective manner, while minimizing treatment-related toxicities.

## Molecular Pathogenesis of Rectal Cancer

While the successive genomic alterations that underlie the adenoma to carcinoma sequence remain the framework for our understanding the process of tumorigenesis in CRC (51, 52), recent improvements in sequencing technology have recognized genetic differences between colon and rectal cancers.

### Genomic Instability

It has been appreciated for some time that genomic instability facilitates the multistep progression of CRC (53), and LARC is remarkable for its association with genomic instability. This process results from three different molecular pathways: chromosomal instability (CIN), microsatellite instability (MSI), and CpG island methylator phenotype (CIMP) (54, 55). Understanding the pathway to tumorigenesis has significant clinical ramifications for LARC management, with potential implications for screening and surveillance, response to neoadjuvant and adjuvant therapies, and the selection for targeted therapies.

### Chromosomal Instability Pathway

CIN is the most frequent cause of genomic instability in CRC, occurring in 70% of sporadic CRC, with increasing prevalence in left-sided CRC, including rectal cancer. CIN refers to the gains and losses of whole chromosomes with resultant to aneuploidy, amplifications and loss of heterozygosity (56). This phenotype is usually secondary to alterations in mechanisms that ensure the fidelity of chromosomal segregation (57). These karyotypic abnormalities occur in combination with the accumulation of the “classic” driver mutations in CRC such as APC, KRAS, and SMAD4 (57). CIN in particular has been associated with early-onset CRC, whose incidence has been climbing in recent decades, predominantly in the left colon and rectum (2, 58).

### Microsatellite Instability

In between 10 and 20% of CRC patients, defects in the mismatch repair (MMR) proteins lead to CRC displaying MSI (59). Microsatellites are repetitive sequences distributed throughout the genome that consist of nucleotide repeats that are more frequently copied incorrectly in the presence of a deficient MMR system (dMMR) (60). The resultant MSI phenotype results in numerous frameshift mutations in coding and non-coding microsatellites with neoantigen formation, a “hypermutator phenotype,” and an enhanced local immune response. Consequently, MSI or dMMR in this paper refers interchangeably to MSI CRC by PCR or dMMR tumors by immunohistochemistry. Diagnosis of MSI is via PCR amplification. Alternatively, immunohistochemistry can confirm the presence or absence of MMR proteins (59). MSI is less frequently found in rectal cancer. It primarily occurs in right-sided CRC and is associated with poorly differentiated tumors, a high mucinous component, numerous tumor-infiltrating lymphocytes (TILs), and with the presence of a “Crohn's-like” host response (61). A subset of MSI CRC is caused by autosomal dominant mutations in the DNA MMR system termed Lynch syndrome (59). Lynch syndrome is the most common heritable cancer predisposition syndrome (62).

### CpG Island Methylator Phenotype

CIMP occurs due to the “serrated pathway.” It is characterized by DNA hypermethylation at specific regulatory sites, enriched in CpG motifs (CpG islands) in the promoter regions of tumor suppressor genes, thus leading to transcriptional silencing (63). There is some overlap between CIMP and sporadic MSI cancers owing to their association with methylation of the MLH1 promoter and an activating *BRAF* mutation (64). It is observed in about 15% of tumors (51), particularly right-sided CRC, and is infrequent in rectal tumors (65).

### CMS Subtypes

In an effort to refine the molecular genetic classification of CRC, an international consortium of experts have outlined a CMS classification based on results from six independent transcriptomic-based studies (66). Here, the CIN phenotype was subdivided into three further CMSs, each with distinguishing features: CMS2 (“canonical”), epithelial, marked WNT and MYC signaling activation; CMS3 (“metabolic”), epithelial and evident metabolic dysregulation; and CMS4 (“mesenchymal”), prominent transforming growth factor-β activation, stromal invasion, and angiogenesis (66). MSI or mismatch repair deficient (dMMR) tumors, on the other hand, represented in the CMS1 (“microsatellite instability, hypermutated, immune”) subtype, occurs when there is deficiency in MMR proteins, generally caused by sporadic epigenetic silencing (e.g., by hypermethylation) or by constitutional mutations (e.g., in Lynch syndrome). Samples with mixed features (13%) are proposed to represent a transition phenotype or intratumoral heterogeneity. Although the CMS classification does not yet impact on colon or rectal cancer management, it is hoped that this robust classification system may provide the basis for future clinical stratification and subtype-based targeted interventions.

## Clinical Implications of Rectal Cancer Molecular Pathogenesis

### Microsatellite Instability

Although MSI is a well-established biomarker in CRC, its significance in rectal cancer specifically was until recently uncertain. This is mainly a result of the relative infrequency of MSI rectal cancer, accounting for just 2–15% of all MSI CRC (67, 68). However, it has long been appreciated that the occurrence of MSI in LARC is highly predictive of Lynch syndrome (65, 67, 69). Consequently, whenever MSI or dMMR in LARC is recognized, diagnostic constitutional mutation analysis should be undertaken, either via Sanger sequencing of MMR genes (70) or as part of a next-generation sequencing multiplex gene panel, regardless of BRAF mutation status (69, 71).

Even once the diagnosis has been made, the optimal treatment of Lynch-associated LARC remains unclear. For instance, unlike in MSI colon cancer, deficiency in the MSH2/MSH6 heterodimer is the most common pattern of MMR protein loss (69). Mutations in these MMR genes have been associated with increased frequencies of extracolonic cancers (72). In the biggest analysis of MSI rectal cancer to date, 23% of participants developed an extracolonic malignancy, contributing to 45% of mortalities (69). Thus, although evidence of benefit is limited (73–75), surveillance for multiorgan cancer development, ideally as part of clinical trials, is important. The extent of bowel resection also remains controversial. Optimal oncologic outcome must be balanced against sphincter preservation and quality of life (76, 77). The incidence of metachronous CRC was observed to be 19% at 10 years (78–81) with an associated up to six-fold excess mortality (78). For this reason, an aggressive management approach is suggested in recurrent and/or metachronous disease.

There remains a scarceness of long-term oncological outcome data for MSI rectal cancer specifically; this is despite evidence that MSI rectal and colon cancers have distinct biology (82). Thus, the prognosis for LARC with MSI treated with conventional multimodal therapy was until recently unclear (83). Unlike MSI colon cancers, where there is a preponderance of right-sided cancers in aging females, over a third of MSI CRC in Asian men developed in the rectum (68). MSI rectal cancer displays other distinctive features, with a significant mucinous component (84) and a decreased occurrence of MLH1 promoter hypermethylation and expression of BRAF mutations unlike Lynch dMMR colon cancer, which has no hMLH1promoter hypermethylation (85, 86). The reduced incidence of BRAF mutations may in part explain the improved prognosis of MSI LARC, with 5-year OS and DFS being reported as 90.6 (69) and 70% (87), and 5-year OS of 50% for disease with distant metastatic spread (88).

The development of predictive biomarkers to appropriately select patients for the various treatment modalities for LARC has proved challenging. Despite the general agreement that MSI predicts poor response to adjuvant 5-fluouracil–based chemotherapy (89), its impact on response to 5-fluouracil given as part of nCRT is controversial (90). In MSI with LARC, an increased sensitivity to radiotherapy has been observed in preclinical studies (91–93). Initial underpowered series with little clinicopathologic detail and considerable heterogeneity have described pCR rates of between 0 and 60% (59). More recently, in the largest ever clinical series, tumor regression was demonstrated to be excellent, with a pCR rate of 27.6 vs. 18% in patients with MSI and MSS LARC, respectively (69, 87).

Another consideration in rectal cancer undergoing nCRT is that in cases of pCR, no residual tumor may be available for further MMR or MSI testing, and in some instances, nCRT has been shown to alter the MMR protein status of the tumor (94). For this reason, testing of the pre-treatment biopsy should be undertaken to ensure a source of suitable, reliable testing material is available (95). Although pCR may also occur with chemotherapy alone, MSI LARC appears to have more heterogeneous responses to induction chemotherapy, with chemoresistance being reported in some instances (96). However, evidence suggests that advanced dMMR tumors may respond remarkably well to immunotherapy (97, 98). Emerging trials evaluating the combination of immunotherapy and nCRT in patients with MSI LARC (NCT02948348, NCT03038477, and NCT03854799) based on the preliminary results of such an approach in the neoadjuvant and metastatic disease settings are currently underway (99, 100).

### Chromosomal Instability Pathway

In contrast to MSI LARC, CIN, which is much more common in rectal tumors, displays much less immunogenicity. However, the copy number alterations that are a feature of CIN LARC may generate possible targets for therapy such as HER2 amplification, outlined as part of the following section on targeted therapies.

### Specific Mutations

Both APC and TP53 mutations are more common in rectal cancer (101, 102). Conversely, KRAS mutations are less common, as are mutations in BRAF (102). In clinical practice, these differences in genomic alterations determine suitability for treatment with targeted therapies and may affect response to multimodality therapy and patterns of metastatic spread (103–105). Aside from increasing the interval from the end of nCRT to surgery (43, 106), there are few pathological, molecular, or immunologic features consistently associated with pCR after nCRT (107). Studies included in a recent systematic review evaluating a number of molecular markers, including gene signatures by microarray, single-nucleotide polymorphisms, TP53 and mutations in KRAS, and proteomic profiles failed to find any single reliable predictor of pCR (107). However, a more recent multicenter study of almost 300 patients with stage II/III rectal cancer observed pCR rates in KRAS wild-type and KRAS mutant tumors of 34 and 15%, respectively (108). In multivariable analysis, KRAS mutation remained independently associated with a lower pCR rate.

When metastases occur, rectal tumors more frequently metastasize to the lungs and bones, and less frequently metastasize to the gynecologic organs or the omentum and peritoneum in comparison with colon cancers (102). The reduced incidence of peritoneal involvement has been hypothesized to be a result of the relative infrequency of MSI and BRAF mutations in LARC. There is an increased likelihood of lung metastasis in distal rectal cancers despite a reduced incidence of KRAS mutations, a molecular feature often associated with lung metastasis (104, 105). This is primarily caused by their associated venous drainage via the inferior rectal vein, thus bypassing the portal venous system, although treatment nCRT further increases the likelihood of lung metastasis (103, 109).

### Consensus Molecular Subtypes

Although the CMS classification offers clues into the transcriptional program of CRC, this has not yet had a major impact on clinical management. However, in the original paper, the CMS4 (“mesenchymal”) subgroup was associated with worse DFS and OS, whereas CMS1 (“microsatellite instability, hypermutated, immune”) was associated with worse survival after disease recurrence (66). These molecular subgroups are distributed unequally between rectal and colon cancers. This raises the possibility that the results of future clinical trials with CMS subtype-based targeted interventions may be disproportionately affected by primary tumor location.

## Potential Targeted Therapies

Rectal cancers generally display wild-type RAS genes and are thus often candidates for targeted therapy with EGFR inhibitors. In contrast, RAS mutations are more common in right-sided tumors and these mutations are associated with resistance to EGFR inhibitors (110). The expression of the EGFR ligands, amphiregulin and epiregulin, varies across by primary tumor location and may also predict response to this type of targeted therapy (111). EGFR inhibitors have also been tested in LARC, but when used as an adjunct to standard therapies have not been able to enhance the pCR rate (10).

Because of the excessive rate of copy number alterations, gene amplifications are more common in left-sided CRC. The amplification of the receptor tyrosine-protein kinase HER2 is an emerging target. The prevalence of HER2 amplification was demonstrated, in a recent retrospective study, to be significantly increased in rectal cancer (10.4%) compared with left-sided (3.6%) and right-sided CRC (2.9%) (112), while another large retrospective study (*n* = 717) demonstrated that HER2 overexpression by immunohistochemistry was found in 16% of rectal cancers and was associated with worse 5-year OS vs. HER2-negative patients (63.5 vs. 73.9%, *P* = 0.013) (113). Contemporary studies of dual HER2-targeted therapy with the combinations of trastuzumab–pertuzumab and trastuzumab–lapatinib have demonstrated promising initial results in metastatic HER2-amplified CRC, rates of response in the region of approximately 30% (114, 115).

Mutations in BRAF, including the V600E “hotspot,” are rare in rectal cancer (<1%). BRAF mutations are related to worse prognosis in CRC and a lack of benefit from EGFR inhibitors (116, 117). Thus, despite its relative infrequency, testing for mutations in BRAF is important to guide the use of EGFR inhibitors and of possibly BRAF-targeted agents. At present, there are no FDA-approved anti-BRAF drugs; however, combinations of selective RAF and EGFR inhibitors have shown encouraging initial results and the National Comprehensive Cancer Network recommend a combination of RAF inhibitor vemurafenib with cetuximab and the chemotherapy agent irinotecan for BRAF V600E-mutated CRC owing to its improved progression-free survival compared with regimens without RAF inhibition (118). Moreover, the combination of the RAF inhibitor encorafenib, the mitogen-activated protein kinase [MEK (the protein target of BRAF)] inhibitor binimetinib, and cetuximab was recently granted FDA approval for the treatment of BRAF V600E-mutated CRC if resistant to initial regimens (119).

As noted previously, up to 5% of rectal cancer displays the CMS1 (“microsatellite instability, hypermutated, immune”) subtype, most frequently caused by mutations in the MMR genes. This displays upregulation of various immune checkpoints including programmed death (PD-1) and cytotoxic T lymphocyte–associated protein 4 (CTLA4) that act to downgrade the host inflammatory response in an effort to limit immune-mediated tissue damage. This raises the possibility of immune checkpoint inhibition therapy for MSI LARC. The rates of response to single agent anti-PD1 inhibitors varies between 30 and 50% and combination management with dual immune checkpoint inhibition achieves response rates of over 50% (98, 100). Moreover, these strategies have been associated with durable benefit in responders (105).

## Immunological Landscape of Rectal Cancer

It is well-recognized that the progression and recurrence of cancer is not just regulated by the genomic mutations inherent to malignant cells but also by host and immunological responses (120). Various mechanisms in the TIME may stimulate a pro-tumorigenic environment and disease progression. The recruitment of immunoregulatory cells and upregulation of inhibitory molecules (e.g., T regulatory cells, natural killer cells, type 2 macrophages, dendritic cells, myeloid-derived suppressor cells, and other cancer-associated cell types), as well as the downregulation of antigen presentation all facilitate immune evasion (120). Furthermore, the promotion of glycolytic and anabolic pathways often leads to an acidic and anaerobic environment with resultant T-cell exhaustion (121). This may be exacerbated by upregulation of various immune checkpoints PD-1 and CTLA4 that act to downgrade the host inflammatory response in an effort to limit immune-mediated tissue damage (122). Loss of MHC class I and II proteins from cell surfaces that are required for antigen presentation to T cells represents another immunosuppressive mechanism (123, 124).

In contrast, clinicopathological evidence demonstrates that effector T cells in the TIME are key effectors of outcome in CRC. Consequently, patients with higher TIL numbers have improved survival (125). Based on this principle, Galon et al. (126, 127) have developed a TIME-based immunocytochemical score, the Immunoscore (HalioDx, Marseille, France), and this has been shown to be a superior prognosticator to even TNM staging (128). From a systemic perspective, an inflammatory marker that is increasingly used is the neutrophil/lymphocyte ratio (NLR). A recent meta-analysis established that a raised NLR correlated with significantly reduced OS and progression-free survival rates in CRC (129). Markers of systemic inflammation, including NLR, is also a predictor of outcome in patients undergoing nCRT for LARC (130). Furthermore, NLR has been shown to be inversely associated with the vigorous antitumor TIL infiltrate associated with MSI CRC (131). Consequently, NLR may represent a more cost-effective and clinical useful marker than newer and ostensibly more sophisticated immune markers.

### Potential Therapeutic Implications of the Different Immune Signatures

Although immune cell quantification approaches such as the Immunoscore give a phenotypic depiction of the TIME, the comparative influences of germline, somatic, and epigenetic variations in CRC immune signatures, including TIL numbers, have yet to be fully elucidated. It is evident that somatic mutational factors in isolation are not adequate to explain this variability. Recent evidence suggests that right- and left-sided CRC, including rectal cancer, have distinctive immunological phenotypes with several biological and clinical distinctions including embryological origin, vascular supply, physiologic function, and local microbiota (132). This may affect somatic mutations and immunological landscapes between the disease locations as well as alter response to therapy.

There is increased infiltration of antitumor CD56^bright^ NK cell subpopulations in rectal cancer and other left-sided CRC (133). This may explain why left-sided CRC with wild-type RAS demonstrates better response to EGFR inhibitors than that which occurs in patients with CRC from other primary tumor locations (134). PD-1 receptors have also been detected on activated NK cells (135, 136), suggesting that these tumors may respond to immune checkpoint inhibition. Therefore, the combination of anti-PD-L1 and EGFR inhibitors may be a potential therapy for RAS wild-type rectal cancer. In contrast, there is increased CD8^+^ TIL infiltration, cytotoxic activity, interferon-γ signature, and antigen processing machinery in right-sided CRC. This immune phenotype is consistent with the higher tumor mutational burden and incidence of MSI in right-sided CRC (137). However, this favorable CD8^+^ TIL-mediated antitumor reaction might be impeded by increased concentrations of VEGF-A, which not only mediates immune tolerance but also restricts TIL numbers (133). These results may explain the paradoxical worse prognosis of right-sided CRC despite more efficient CD8^+^ T-cell infiltration (125) and why right-sided CRC respond better to anti-VEGF therapy than rectal tumors (138). Right-sided CRC also display generally higher levels of PD-1/PD-L1 owing to their immunogenicity (139). Thus, co-targeting angiogenesis and immune checkpoints may represent a promising therapeutic strategy for right-sided CRC but not rectal cancer (140).

### The Important Role of the Host Immune System in Radiotherapy Response

Preclinical (141) immunological and genomic (142) studies have demonstrated the critical role of the host immune system in promoting nCRT-induced tumor regression. The presence of TILs in pretreatment biopsies may predict a better response to nCRT in rectal cancer (143, 144). It has been demonstrated that there are important roles for TILs, neoantigens, and PD-1/LAG3 signaling in rectal tumor response to nCRT (145). For this reason, these immune checkpoints may be therapeutic targets to improve nCRT-induced tumor regression in rectal cancer (145, 146). Several clinical trials are ongoing to test whether combining immune checkpoint inhibitors with nCRT may enhance pCR rates and tumor regression (NCT03127007, NCT03102047, and NCT02948348). Preliminary data from a multicenter phase Ib–II study (147) of nivolumab (an anti-PD-1 antibody) monotherapy as an adjunct to conventional nCRT demonstrated a high pCR rate, but the overall participant numbers were small. However, the hypothesis for such a strategy is supported by the evidence that hypermutated rectal cancers, caused by MMR or POLE mutations, have a good prognosis and excellent response to nCRT (69, 145).

## Conclusion

Rectal cancer has many important clinicopathologic differences from colon cancer and presents a unique challenge requiring careful evaluation and management involving pathologists, radiologists, surgeons, and radiation and medical oncologists. The current TNM classification stratifies rectal cancer patients by stage, ultimately impacting whether patients receive neoadjuvant therapy. However, there is considerable heterogeneity in terms of response rates and outcomes within stages. Therefore, a multimodality approach is crucial in providing appropriate and effective treatment to rectal cancer patients. An understanding of the pathological, molecular genetics, and immunological features underlying CRC tumorigenesis and progression provides a further framework to evaluate the disease and its management. Delivery of personalized multimodal care for LARC in the coming years will require sophisticated prognostic modeling that accounts for this heterogeneity as well as consideration of the socioeconomic and healthcare context in which this care is delivered.

## Author Contributions

All authors contributed significantly to all aspects of the paper.

## Conflict of Interest

The authors declare that the research was conducted in the absence of any commercial or financial relationships that could be construed as a potential conflict of interest.

## References

[1] Ferlay J Colombet M Soerjomataram I Dyba T Randi G Bettio M. Cancer incidence and mortality patterns in Europe: estimates for 40 countries and 25 major cancers in 2018. Eur J Cancer. (2018) 103:356–87. 10.1016/j.ejca.2018.07.005 30100160

[2] Segev L Kalady MF Church JM. Left-sided dominance of early-onset colorectal cancers: a rationale for screening flexible sigmoidoscopy in the young. Dis Colon Rectum. (2018) 61:897–902. 10.1097/DCR.0000000000001062 29771800

[3] Kapiteijn E Marijnen CA Nagtegaal ID Putter H Steup WH Wiggers T. Preoperative radiotherapy combined with total mesorectal excision for resectable rectal cancer. N Engl J Med. (2001) 345:638–46. 10.1056/NEJMoa010580 11547717

[4] Peeters KC Marijnen CA Nagtegaal ID Kranenbarg EK Putter H Wiggers T. The TME trial after a median follow-up of 6 years: increased local control but no survival benefit in irradiated patients with resectable rectal carcinoma. Ann Surg. (2007) 246:693–701. 10.1097/01.sla.0000257358.56863.ce 17968156

[5] Sauer R Liersch T Merkel S Fietkau R Hohenberger W Hess C. Preoperative versus postoperative chemoradiotherapy for locally advanced rectal cancer: results of the German CAO/ARO/AIO-94 randomized phase III trial after a median follow-up of 11 years. J Clin Oncol. (2012) 30:1926–33. 10.1200/JCO.2011.40.1836 22529255

[6] Sauer R Becker H Hohenberger W Rödel C Wittekind C Fietkau R. Preoperative versus postoperative chemoradiotherapy for rectal cancer. N Engl J Med. (2004) 351:1731–40. 10.1056/NEJMoa040694 15496622

[7] Glehen O Chapet O Adham M Nemoz JC Gerard JP. Long-term results of the Lyons R90-01 randomized trial of preoperative radiotherapy with delayed surgery and its effect on sphincter-saving surgery in rectal cancer. Br J Surg. (2003) 90:996–8. 10.1002/bjs.4162 12905554

[8] Marks KM West NP Morris E Quirke P. Clinicopathological, genomic and immunological factors in colorectal cancer prognosis. Br J Surg. (2018) 105:e99–e109. 10.1002/bjs.10756 29341159 PMC7938848

[9] Mondaca S Yaeger R. Genetics of rectal cancer and novel therapies: primer for radiologists. Abdom Radiol. (2019) 44:3743–50. 10.1007/s00261-019-02051-x 31073721 PMC6824989

[10] Clifford R Govindarajah N Parsons JL Gollins S West NP Vimalachandran D. Systematic review of treatment intensification using novel agents for chemoradiotherapy in rectal cancer. Br J Surg. (2018) 105:1553–72. 10.1002/bjs.10993 30311641 PMC6282533

[11] Zaborowski A Stakelum A Winter DC. Systematic review of outcomes after total neoadjuvant therapy for locally advanced rectal cancer. Br J Surg. (2019) 106:979–87. 10.1002/bjs.11171 31074508

[12] Snelgrove RC Subendran J Jhaveri K Thipphavong S Cummings B Brierley J. Effect of multidisciplinary cancer conference on treatment plan for patients with primary rectal cancer. Dis Colon Rectum. (2015) 58:653–8. 10.1097/DCR.0000000000000390 26200679

[13] Taylor FG Quirke P Heald RJ Moran BJ Blomqvist L Swift IR. Preoperative magnetic resonance imaging assessment of circumferential resection margin predicts disease-free survival and local recurrence: 5-year follow-up results of the MERCURY study. J Clin Oncol. (2014) 32:34–43. 10.1200/JCO.2012.45.3258 24276776

[14] Chand M Swift RI Tekkis PP Chau I Brown G. Extramural venous invasion is a potential imaging predictive biomarker of neoadjuvant treatment in rectal cancer. Br J Cancer. (2014) 110:19–25. 10.1038/bjc.2013.603 24300971 PMC3887281

[15] Sohn B Lim JS Kim H Myoung S Choi J Kim NK. MRI-detected extramural vascular invasion is an independent prognostic factor for synchronous metastasis in patients with rectal cancer. Eur Radiol. (2015) 25:1347–55. 10.1007/s00330-014-3527-9 25500963

[16] Jhaveri KS Hosseini-Nik H Thipphavong S Assarzadegan N Menezes RJ Kennedy ED. MRI detection of extramural venous invasion in rectal cancer: correlation with histopathology using elastin stain. AJR Am J Roentgenol. (2016) 206:747–55. 10.2214/AJR.15.15568 26933769

[17] Kim DJ Kim JH Lim JS Yu JS Chung JJ Kim MJ. Restaging of rectal cancer with MR imaging after concurrent chemotherapy and radiation therapy. Radiographics. (2010) 30:503–16. 10.1148/rg.302095046 20228331

[18] Hanly AM Ryan EM Rogers AC McNamara DA Madoff RD Winter DC. Multicenter evaluation of rectal cancer ReImaging pOst neoadjuvant (MERRION) therapy. Ann Surg. (2014) 259:723–7. 10.1097/SLA.0b013e31828f6c91 23744576

[19] van der Paardt MP Zagers MB Beets-Tan RG Stoker J Bipat S. Patients who undergo preoperative chemoradiotherapy for locally advanced rectal cancer restaged by using diagnostic MR imaging: a systematic review and meta-analysis. Radiology. (2013) 269:101–12. 10.1148/radiol.13122833 23801777

[20] Kuo LJ Chiou JF Tai CJ Chang CC Kung CH Lin SE. Can we predict pathologic complete response before surgery for locally advanced rectal cancer treated with preoperative chemoradiation therapy? Int J Colorectal Dis. (2012) 27:613–21. 10.1007/s00384-011-1348-8 22080392

[21] Chand M Evans J Swift RI Tekkis PP West NP Stamp G. The prognostic significance of postchemoradiotherapy high-resolution MRI and histopathology detected extramural venous invasion in rectal cancer. Ann Surg. (2015) 261:473–9. 10.1097/SLA.0000000000000848 25243543

[22] Patel U Brown G Machado I Santos-Cores J Pericay C Ballesteros E. MRI assessment and outcomes in patients receiving neoadjuvant chemotherapy only for primary rectal cancer: long-term results from the GEMCAD 0801 trial. Ann Oncol. (2017) 28:344–53. 10.1093/annonc/mdw616 28426108

[23] Lee ES Kim MJ Park SC Hur BY Hyun JH Chang HJ. Magnetic resonance imaging-detected extramural venous invasion in rectal cancer before and after preoperative chemoradiotherapy: diagnostic performance and prognostic significance. Eur Radiol. (2018) 28:496–505. 10.1007/s00330-017-4978-6 28786006

[24] Nagtegaal ID Odze RD Klimstra D Paradis V Rugge M Schirmacher P. The 2019 WHO classification of tumours of the digestive system. Histopathology. (2019) 76:182–8. 10.1111/his.13975 31433515 PMC7003895

[25] van Gijn W Marijnen CA Nagtegaal ID Kranenbarg EM Putter H Wiggers T. Preoperative radiotherapy combined with total mesorectal excision for resectable rectal cancer: 12-year follow-up of the multicentre, randomised controlled TME trial. Lancet Oncol. (2011) 12:575–82. 10.1016/S1470-2045(11)70097-3 21596621

[26] Creavin B Kelly ME Ryan E Winter DC. Meta-analysis of the impact of surgical approach on the grade of mesorectal excision in rectal cancer. Br J Surg. (2017) 104:1609–19. 10.1002/bjs.10664 29044484

[27] Nagtegaal ID Quirke P. What is the role for the circumferential margin in the modern treatment of rectal cancer? J Clin Oncol. (2008) 26:303–12. 10.1200/JCO.2007.12.7027 18182672

[28] Ueno H Murphy J Jass JR Mochizuki H Talbot IC. Tumour 'budding' as an index to estimate the potential of aggressiveness in rectal cancer. Histopathology. (2002) 40:127–32. 10.1046/j.1365-2559.2002.01324.x 11952856

[29] Rogers AC Gibbons D Hanly AM Hyland JM O'Connell PR Winter DC. Prognostic significance of tumor budding in rectal cancer biopsies before neoadjuvant therapy. Mod Pathol. (2014) 27:156–62. 10.1038/modpathol.2013.124 23887296

[30] Rogers AC Winter DC Heeney A Gibbons D Lugli A Puppa G. Systematic review and meta-analysis of the impact of tumour budding in colorectal cancer. Br J Cancer. (2016) 115:831–40. 10.1038/bjc.2016.274 27599041 PMC5046217

[31] Lugli A Kirsch R Ajioka Y Bosman F Cathomas G Dawson H. Recommendations for reporting tumor budding in colorectal cancer based on the international tumor budding consensus conference (ITBCC) (2016). Mod Pathol. (2017) 30:1299–1311. 10.1038/modpathol.2017.46 28548122

[32] Jass JR. Classification of colorectal cancer based on correlation of clinical, morphological and molecular features. Histopathology. (2007) 50:113–30. 10.1111/j.1365-2559.2006.02549.x 17204026

[33] Hogan J Chang KH Duff G Samaha G Kelly N Burton M. Lymphovascular invasion: a comprehensive appraisal in colon and rectal adenocarcinoma. Dis Colon Rectum. (2015) 58:547–55. 10.1097/DCR.0000000000000361 25944426

[34] Jass JR Love SB Northover JM. A new prognostic classification of rectal cancer. Lancet. (1987) 1:1303–6. 10.1016/S0140-6736(87)90552-6 2884421

[35] Messenger DE Driman DK Kirsch R. Developments in the assessment of venous invasion in colorectal cancer: implications for future practice and patient outcome. Hum Pathol. (2012) 43:965–73. 10.1016/j.humpath.2011.11.015 22406362

[36] Liebig C Ayala G Wilks J Verstovsek G Liu H Agarwal N. Perineural invasion is an independent predictor of outcome in colorectal cancer. J Clin Oncol. (2009) 27:5131–7. 10.1200/JCO.2009.22.4949 19738119 PMC2773472

[37] Ceyhan GO Liebl F Maak M Schuster T Becker K Langer R. The severity of neural invasion is a crucial prognostic factor in rectal cancer independent of neoadjuvant radiochemotherapy. Ann Surg. (2010) 252:797–804. 10.1097/SLA.0b013e3181fcab8d 21037435

[38] Bouquot M Creavin B Goasguen N Chafai N Tiret E Andre T. Prognostic value and characteristics of N1c colorectal cancer. Colorectal Dis. (2018) 20:O248–O255. 10.1111/codi.14289 29894583

[39] Edge SB Compton CC. The American joint committee on cancer: the 7th edition of the AJCC cancer staging manual and the future of TNM. Ann Surg Oncol. (2010) 17:1471–4. 10.1245/s10434-010-0985-4 20180029

[40] Ueno H Hashiguchi Y Shimazaki H Shinto E Kajiwara Y Nakanishi K. Peritumoral deposits as an adverse prognostic indicator of colorectal cancer. Am J Surg. (2014) 207:70–77. 10.1016/j.amjsurg.2013.04.009 24112678

[41] Martin ST Heneghan HM Winter DC. Systematic review and meta-analysis of outcomes following pathological complete response to neoadjuvant chemoradiotherapy for rectal cancer. Br J Surg. (2012) 99:918–28. 10.1002/bjs.8702 22362002

[42] Kalady MF de Campos-Lobato LF Stocchi L Geisler DP Dietz D Lavery IC. Predictive factors of pathologic complete response after neoadjuvant chemoradiation for rectal cancer. Ann Surg. (2009) 250:582–9. 10.1097/SLA.0b013e3181b91e63 19710605

[43] Ryan EJ O'Sullivan DP Kelly ME Syed AZ Neary PC O'Connell PR. Meta-analysis of the effect of extending the interval after long-course chemoradiotherapy before surgery in locally advanced rectal cancer. Br J Surg. (2019) 106:1298–310. 10.1002/bjs.11220 31216064

[44] Lefevre JH Mineur L Kotti S Rullier E Rouanet P de Chaisemartin C. Effect of interval (7 or 11 weeks) between neoadjuvant radiochemotherapy and surgery on complete pathologic response in rectal cancer: a multicenter, randomized, controlled trial (GRECCAR-6). J Clin Oncol. (2016) 34:3773–80. 10.1200/JCO.2016.67.6049 27432930

[45] Garcia-Aguilar J Chow OS Smith DD Marcet JE Cataldo PA Varma MG. Effect of adding mFOLFOX6 after neoadjuvant chemoradiation in locally advanced rectal cancer: a multicentre, phase 2 trial. Lancet Oncol. (2015) 16:957–66. 10.1016/S1470-2045(15)00004-2 26187751 PMC4670237

[46] Habr-Gama A Perez RO Nadalin W Sabbaga J Ribeiro U Jr e Sousa AHS Jr. Operative versus nonoperative treatment for stage 0 distal rectal cancer following chemoradiation therapy: long-term results. Ann Surg. (2004) 240:711–7. 10.1097/01.sla.0000141194.27992.32 15383798 PMC1356472

[47] van der Valk MJM Hilling DE Bastiaannet E Meershoek-Klein Kranenbarg E Beets GL Figueiredo NL. Long-term outcomes of clinical complete responders after neoadjuvant treatment for rectal cancer in the international watch & wait database (IWWD): an international multicentre registry study. Lancet. (2018) 391:2537–45. 10.1016/S0140-6736(18)31078-X 29976470

[48] van den Ende RPJ Peters FP Harderwijk E Rütten H Bouwmans L Berbee M. Radiotherapy quality assurance for mesorectum treatment planning within the multi-center phase II STAR-TReC trial: dutch results. Radiat Oncol. (2020) 15:41. 10.1186/s13014-020-01487-6 32070386 PMC7027245

[49] Brouquet A Bachet JB Huguet F Karoui M Artru P Sabbagh C. NORAD01-GRECCAR16 multicenter phase III non-inferiority randomized trial comparing preoperative modified FOLFIRINOX without irradiation to radiochemotherapy for resectable locally advanced rectal cancer (intergroup FRENCH-GRECCAR- PRODIGE trial). BMC Cancer. (2020) 20:485. 10.1186/s12885-020-06968-1 32471382 PMC7257230

[50] Rouanet P Rullier E Lelong B Maingon P Tuech JJ Pezet D. Tailored treatment strategy for locally advanced rectal carcinoma based on the tumor response to induction chemotherapy: preliminary results of the french phase II multicenter GRECCAR4 trial. Dis Colon Rectum. (2017) 60:653–63. 10.1097/DCR.0000000000000849 28594714

[51] Markowitz SD Bertagnolli MM. Molecular origins of cancer: molecular basis of colorectal cancer. N Engl J Med. (2009) 361:2449–60. 10.1056/NEJMra0804588 20018966 PMC2843693

[52] Fearon ER Vogelstein B. A genetic model for colorectal tumorigenesis. Cell. (1990) 61:759–67. 10.1016/0092-8674(90)90186-I 2188735

[53] Hanahan D Weinberg RA. Hallmarks of cancer: the next generation. Cell. (2011) 144:646–74. 10.1016/j.cell.2011.02.013 21376230

[54] Cunningham D Atkin W Lenz HJ Lynch HT Minsky B Nordlinger B. Colorectal cancer. Lancet. (2010) 375:1030–47. 10.1016/S0140-6736(10)60353-4 20304247

[55] Ogino S Chan AT Fuchs CS Giovannucci E. Molecular pathological epidemiology of colorectal neoplasia: an emerging transdisciplinary and interdisciplinary field. Gut. (2011) 60:397–411. 10.1136/gut.2010.217182 21036793 PMC3040598

[56] Lengauer C Kinzler KW Vogelstein B. Genetic instabilities in human cancers. Nature. (1998) 396:643–9. 10.1038/25292 9872311

[57] Pino MS Chung DC. The chromosomal instability pathway in colon cancer. Gastroenterology. (2010) 138:2059–72. 10.1053/j.gastro.2009.12.065 20420946 PMC4243705

[58] Perea J Rueda D Canal A Rodriguez Y Alvaro E Osorio I. Age at onset should be a major criterion for subclassification of colorectal cancer. J Mol Diagn. (2014) 16:116–26. 10.1016/j.jmoldx.2013.07.010 24184227

[59] Ryan E Sheahan K Creavin B Mohan HM Winter DC. The current value of determining the mismatch repair status of colorectal cancer: a rationale for routine testing. Crit Rev Oncol Hematol. (2017) 116:38–57. 10.1016/j.critrevonc.2017.05.006 28693799

[60] Ogino S Goel A. Molecular classification and correlates in colorectal cancer. J Mol Diagn. (2008) 10:13–27. 10.2353/jmoldx.2008.070082 18165277 PMC2175539

[61] Jenkins MA Hayashi S O'Shea AM Burgart LJ Smyrk TC Shimizu D. Pathology features in bethesda guidelines predict colorectal cancer microsatellite instability: a population-based study. Gastroenterology. (2007) 133:48–56. 10.1053/j.gastro.2007.04.044 17631130 PMC2933045

[62] Vasen HF Moslein G Alonso A Bernstein I Bertario L Blanco I. Guidelines for the clinical management of lynch syndrome (hereditary non-polyposis cancer). J Med Genet. (2007) 44:353–62. 10.1136/jmg.2007.048991 17327285 PMC2740877

[63] Toyota M Ahuja N Ohe-Toyota M Herman JG Baylin SB Issa JP. CpG island methylator phenotype in colorectal cancer. Proc Natl Acad Sci USA. (1999) 96:8681–6. 10.1073/pnas.96.15.8681 10411935 PMC17576

[64] Kane MF Loda M Gaida GM Lipman J Mishra R Goldman H. Methylation of the hMLH1 promoter correlates with lack of expression of hMLH1 in sporadic colon tumors and mismatch repair-defective human tumor cell lines. Cancer Res. (1997) 57:808–11. 9041175

[65] Sato K Kawazu M Yamamoto Y Ueno T Kojima S Nagae G. Fusion kinases identified by genomic analyses of sporadic microsatellite instability–high colorectal cancers. Clin Cancer Res. (2019) 25:378–89. 10.1158/1078-0432.CCR-18-1574 30279230

[66] Guinney J Dienstmann R Wang X De Reyniès A Schlicker A Soneson C. The consensus molecular subtypes of colorectal cancer. Nat Med. (2015) 21:1350–6. 10.1038/nm.3967 26457759 PMC4636487

[67] Nilbert M Planck M Fernebro E Borg A Johnson A. Microsatellite instability is rare in rectal carcinomas and signifies hereditary cancer. Eur J Cancer. (1999) 35:942–5. 10.1016/S0959-8049(99)00045-3 10533476

[68] Ishikubo T Nishimura Y Yamaguchi K Khansuwan U Arai Y Kobayashi T. The clinical features of rectal cancers with high-frequency microsatellite instability (MSI-H) in Japanese males. Cancer Lett. (2004) 216:55–62. 10.1016/j.canlet.2004.07.017 15500949

[69] de Rosa N Rodriguez-Bigas MA Chang GJ Veerapong J Borras E Krishnan S. DNA mismatch repair deficiency in rectal cancer: benchmarking its impact on prognosis, neoadjuvant response prediction, and clinical cancer genetics. J Clin Oncol. (2016) 34:3039–46. 10.1200/JCO.2016.66.6826 27432916 PMC5012714

[70] Moreira L Balaguer F Lindor N de la Chapelle A Hampel H Aaltonen LA. Identification of Lynch syndrome among patients with colorectal cancer. JAMA. (2012) 308:1555–65. 10.1001/jama.2012.13088 23073952 PMC3873721

[71] Yurgelun MB Allen B Kaldate RR Bowles KR Judkins T Kaushik P. Identification of a variety of mutations in cancer predisposition genes in patients with suspected lynch syndrome. Gastroenterology. (2015) 149:604–13.e620. 10.1053/j.gastro.2015.05.006 25980754 PMC4550537

[72] Stoffel EM Mangu PB Limburg PJ. Hereditary colorectal cancer syndromes: American society of clinical oncology clinical practice guideline endorsement of the familial risk-colorectal cancer: European society for medical oncology clinical practice guidelines. J Oncol Pract. (2015) 11:e437–41. 10.1200/JOP.2015.003665 25829526

[73] Vasen HF Ghorbanoghli Z Bourdeaut F Cabaret O Caron O Duval A. Guidelines for surveillance of individuals with constitutional mismatch repair-deficiency proposed by the European consortium “Care for CMMR-D” (C4CMMR-D). J Med Genet. (2014) 51:283–93. 10.1136/jmedgenet-2013-102238 24556086

[74] Rubenstein JH Enns R Heidelbaugh J Barkun A. American gastroenterological association institute guideline on the diagnosis and management of lynch syndrome. Gastroenterology. (2015) 149:777–82. 10.1053/j.gastro.2015.07.036 26226577

[75] Stoffel EM Kastrinos F. Familial colorectal cancer, beyond lynch syndrome. Clin Gastroenterol Hepatol. (2014) 12:1059–68. 10.1016/j.cgh.2013.08.015 23962553 PMC3926911

[76] Wertzberger BE Sherman SK Byrn JC Differences in short-term outcomes among patients undergoing IPAA with or without preoperative radiation: a national surgical quality improvement program analysis. Dis Colon Rectum. (2014) 57:1188–94. 10.1097/DCR.0000000000000206 25203375 PMC4161052

[77] Coffey JC Winter DC Neary P Murphy A Redmond HP Kirwan WO. Quality of life after ileal pouch-anal anastomosis: an evaluation of diet and other factors using the cleveland global quality of life instrument. Dis Colon Rectum. (2002) 45:30–38. 11786761

[78] Cirillo L Urso ED Parrinello G Pucciarelli S Moneghini D Agostini M. High risk of rectal cancer and of metachronous colorectal cancer in probands of families fulfilling the amsterdam criteria. Ann Surg. (2013) 257:900–4. 10.1097/SLA.0b013e31826bff79 22968081

[79] Kalady MF Lipman J McGannon E Church JM. Risk of colonic neoplasia after proctectomy for rectal cancer in hereditary nonpolyposis colorectal cancer. Ann Surg. (2012) 255:1121–5. 10.1097/SLA.0b013e3182565c0b 22549751

[80] Lee JS Petrelli NJ Rodriguez-Bigas MA. Rectal cancer in hereditary nonpolyposis colorectal cancer. Am J Surg. (2001) 181:207–10. 10.1016/S0002-9610(01)00568-2 11376572

[81] Win AK Parry S Parry B Kalady MF Macrae FA Ahnen DJ. Risk of metachronous colon cancer following surgery for rectal cancer in mismatch repair gene mutation carriers. Ann Surg Oncol. (2013) 20:1829–36. 10.1245/s10434-012-2858-5 23358792 PMC4041733

[82] Andre T de Gramont A Vernerey D Chibaudel B Bonnetain F Tijeras-Raballand A. Adjuvant fluorouracil, leucovorin, and oxaliplatin in stage II to III colon cancer: updated 10-year survival and outcomes according to BRAF mutation and mismatch repair status of the MOSAIC study. J Clin Oncol. (2015) 33:4176–87. 10.1200/JCO.2015.63.4238 26527776

[83] Hong SP Min BS Kim TI Cheon JH Kim NK Kim H. The differential impact of microsatellite instability as a marker of prognosis and tumour response between colon cancer and rectal cancer. Eur J Cancer. (2012) 48:1235–43. 10.1016/j.ejca.2011.10.005 22071131

[84] Takemoto N Konishi F Yamashita K Kojima M Furukawa T Miyakura Y. The correlation of microsatellite instability and tumor-infiltrating lymphocytes in hereditary non-polyposis colorectal cancer (HNPCC) and sporadic colorectal cancers: the significance of different types of lymphocyte infiltration. Jpn J Clin Oncol. (2004) 34:90–98. 10.1093/jjco/hyh018 15067103

[85] Samowitz WS Curtin K Wolff RK Tripp SR Caan BJ Slattery ML. Microsatellite instability and survival in rectal cancer. Cancer Causes Control. (2009) 20:1763–8. 10.1007/s10552-009-9410-3 19669908 PMC2895463

[86] Kuismanen SA Holmberg MT Salovaara R de la Chapelle A Peltomäki P. Genetic and epigenetic modification of MLH1 accounts for a major share of microsatellite-unstable colorectal cancers. Am J Pathol. (2000) 156:1773–9. 10.1016/S0002-9440(10)65048-1 10793088 PMC1876911

[87] Park IJ You YN Agarwal A Skibber JM Rodriguez-Bigas MA Eng C Neoadjuvant treatment response as an early response indicator for patients with rectal cancer. J Clin Oncol. (2012) 30:1770–76. 10.1200/JCO.2011.39.7901 22493423 PMC3383178

[88] Brouquet A Mortenson MM Vauthey JN Rodriguez-Bigas MA Overman MJ Chang GJ Surgical strategies for synchronous colorectal liver metastases in 156 consecutive patients: classic, combined or reverse strategy? J Am Coll Surg. (2010) 210:934–41. 10.1016/j.jamcollsurg.2010.02.039 20510802

[89] Ribic CM Sargent DJ Moore MJ Thibodeau SN French AJ Goldberg RM. Tumor microsatellite-instability status as a predictor of benefit from fluorouracil-based adjuvant chemotherapy for colon cancer. N Engl J Med. (2003) 349:247–57. 10.1056/NEJMoa022289 12867608 PMC3584639

[90] Lim SH Chua W Henderson C Ng W Shin JS Chantrill L. Predictive and prognostic biomarkers for neoadjuvant chemoradiotherapy in locally advanced rectal cancer. Crit Rev Oncol Hematol. (2015) 96:67–80. 10.1016/j.critrevonc.2015.05.003 26032919

[91] Shin J-S Tut TG Yang T Lee CS. Radiotherapy response in microsatellite instability related rectal cancer. Korean J Pathol. (2013) 47:1–8. 10.4132/KoreanJPathol.2013.47.1.1 23482947 PMC3589603

[92] Franchitto A Pichierri P Piergentili R Crescenzi M Bignami M Palitti F. The mammalian mismatch repair protein MSH2 is required for correct MRE11 and RAD51 relocalization and for efficient cell cycle arrest induced by ionizing radiation in G2 phase. Oncogene. (2003) 22:2110–20. 10.1038/sj.onc.1206254 12687013

[93] Barwell J Pangon L Hodgson S Georgiou A Kesterton I Slade T. Biallelic mutation of MSH2 in primary human cells is associated with sensitivity to irradiation and altered RAD51 foci kinetics. J Med Genet. (2007) 44:516–20. 10.1136/jmg.2006.048660 17483304 PMC2597924

[94] Vilkin A Halpern M Morgenstern S Brazovski E Gingold-Belfer R Boltin D. How reliable is immunohistochemical staining for DNA mismatch repair proteins performed after neoadjuvant chemoradiation? Hum Pathol. (2014) 45:2029–36. 10.1016/j.humpath.2014.07.005 25150747

[95] O'Brien O Ryan É Creavin B Kelly ME Mohan HM Geraghty R. Correlation of immunohistochemical mismatch repair protein status between colorectal carcinoma endoscopic biopsy and resection specimens. J Clin Pathol. (2018) 71:631–6. 10.1136/jclinpath-2017-204946 29419411

[96] Cercek A Dos Santos Fernandes G Roxburgh C Ng S Yaeger R Segal N 500PMismatch repair deficient rectal cancer is resistant to induction combination chemotherapy. Ann Oncol. (2018) 29(Suppl. 8):viii150–viii204. 10.1093/annonc/mdy281.047

[97] Le DT Durham JN Smith KN Wang H Bartlett BR Aulakh LK. Mismatch repair deficiency predicts response of solid tumors to PD-1 blockade. Science. (2017) 357:409–13. 10.1126/science.aan6733 28596308 PMC5576142

[98] Le DT Uram JN Wang H Bartlett BR Kemberling H Eyring AD. PD-1 blockade in tumors with mismatch-repair deficiency. N Engl J Med. (2015) 372:2509–20. 10.1056/NEJMoa1500596 26028255 PMC4481136

[99] Chalabi M Fanchi L Van den Berg J Beets G Lopez-Yurda M Aalbers A. Neoadjuvant ipilimumab plus nivolumab in early stage colon cancer. In: C. Zielinski, editor. Annals of Oncology. Oxford: Oxford University Press (2018). p. 731.

[100] Overman MJ Lonardi S Wong KYM Lenz HJ Gelsomino F Aglietta M. Durable clinical benefit with nivolumab plus ipilimumab in DNA mismatch repair-deficient/microsatellite instability-high metastatic colorectal cancer. J Clin Oncol. (2018) 36:773–9. 10.1200/JCO.2017.76.9901 29355075

[101] Comprehensive molecular characterization of human colon and rectal cancer Nature. (2012) 487:330–7. 10.1038/nature11252 22810696 PMC3401966

[102] Yaeger R Chatila WK Lipsyc MD Hechtman JF Cercek A Sanchez-Vega F. Clinical sequencing defines the genomic landscape of metastatic colorectal cancer. Cancer cell. (2018) 33:125–36.e3. 10.1016/j.ccell.2017.12.004 29316426 PMC5765991

[103] Chiang JM Hsieh PS Chen JS Tang R You JF Yeh CY. Rectal cancer level significantly affects rates and patterns of distant metastases among rectal cancer patients post curative-intent surgery without neoadjuvant therapy. World J Surg Oncol. (2014) 12:197. 10.1186/1477-7819-12-197 24980147 PMC4108971

[104] Yaeger R Cowell E Chou JF Gewirtz AN Borsu L Vakiani E. RAS mutations affect pattern of metastatic spread and increase propensity for brain metastasis in colorectal cancer. Cancer. (2015) 121:1195–203. 10.1002/cncr.29196 25491172 PMC4523078

[105] Kemeny NE Chou JF Capanu M Gewirtz AN Cercek A Kingham TP. KRAS mutation influences recurrence patterns in patients undergoing hepatic resection of colorectal metastases. Cancer. (2014) 120:3965–71. 10.1002/cncr.28954 25155157 PMC4574496

[106] Petrelli F Sgroi G Sarti E Barni S. Increasing the interval between neoadjuvant chemoradiotherapy and surgery in rectal cancer: a meta-analysis of published studies. Ann Surg. (2016) 263:458–64. 10.1097/SLA.0000000000000368 24263329

[107] Ryan JE Warrier SK Lynch AC Ramsay RG Phillips WA Heriot AG. Predicting pathological complete response to neoadjuvant chemoradiotherapy in locally advanced rectal cancer: a systematic review. Colorectal Dis. (2016) 18:234–46. 10.1111/codi.13207 26531759

[108] Chow OS Kuk D Keskin M Smith JJ Camacho N Pelossof R. KRAS and combined KRAS/TP53 mutations in locally advanced rectal cancer are independently associated with decreased response to neoadjuvant therapy. Ann Surg Oncol. (2016) 23:2548–55. 10.1245/s10434-016-5205-4 27020587 PMC5047012

[109] Ding P Liska D Tang P Shia J Saltz L Goodman K. Pulmonary recurrence predominates after combined modality therapy for rectal cancer: an original retrospective study. Ann Surg. (2012) 256:111–16. 10.1097/SLA.0b013e31825b3a2b 22664562

[110] Douillard J-Y Oliner KS Siena S Tabernero J Burkes R Barugel M. Panitumumab–FOLFOX4 treatment and RAS mutations in colorectal cancer. N Engl J Med. (2013) 369:1023–34. 10.1056/NEJMoa1305275 24024839

[111] Khambata-Ford S Garrett CR Meropol NJ Basik M Harbison CT Wu S. Expression of epiregulin and amphiregulin and K-ras mutation status predict disease control in metastatic colorectal cancer patients treated with cetuximab. J Clin Oncol. (2007) 25:3230–7. 10.1200/JCO.2006.10.5437 17664471

[112] Seo AN Kwak Y Kim DW Kang SB Choe G Kim WH. HER2 status in colorectal cancer: its clinical significance and the relationship between HER2 gene amplification and expression. PLoS ONE. (2014) 9:e98528. 10.1371/journal.pone.0098528 24879338 PMC4039475

[113] Meng X Huang Z Di J Mu D Wang Y Zhao X. Expression of human epidermal growth factor receptor-2 in resected rectal cancer. Medicine (Baltimore). (2015) 94:e2106. 10.1097/MD.0000000000002106 26632727 PMC5058996

[114] Sartore-Bianchi A Trusolino L Martino C Bencardino K Lonardi S Bergamo F. Dual-targeted therapy with trastuzumab and lapatinib in treatment-refractory, KRAS codon 12/13 wild-type, HER2-positive metastatic colorectal cancer (HERACLES): a proof-of-concept, multicentre, open-label, phase 2 trial. Lancet Oncol. (2016) 17:738–46. 10.1016/S1470-2045(16)00150-9 27108243

[115] Hainsworth JD Meric-Bernstam F Swanton C Hurwitz H Spigel DR Sweeney C. Targeted therapy for advanced solid tumors on the basis of molecular profiles: results from mypathway, an open-label, phase iia multiple basket study. J Clin Oncol. (2018) 36:536–42. 10.1200/JCO.2017.75.3780 29320312

[116] Van Cutsem E Kohne CH Lang I Folprecht G Nowacki MP Cascinu S. Cetuximab plus irinotecan, fluorouracil, and leucovorin as first-line treatment for metastatic colorectal cancer: updated analysis of overall survival according to tumor KRAS and BRAF mutation status. J Clin Oncol. (2011) 29:2011–19. 10.1200/JCO.2010.33.5091 21502544

[117] van Brummelen EMJ de Boer A Beijnen JH Schellens JHM. BRAF Mutations as predictive biomarker for response to anti-EGFR monoclonal antibodies. Oncologist. (2017) 22:864–72. 10.1634/theoncologist.2017-0031 28576857 PMC5507642

[118] Kopetz S McDonough SL Morris VK Lenz H-J Magliocco AM Atreya CE Randomized trial of irinotecan and cetuximab with or without vemurafenib in BRAF-mutant metastatic colorectal cancer (SWOG 1406). J Clin Oncol. (2017) 35(Suppl. 4):520 10.1200/JCO.2017.35.4_suppl.520 PMC8462593 33356422

[119] Cutsem EV Cuyle P-J Huijberts S Yaeger R Schellens JHM Elez E BEACON CRC study safety lead-in (SLI) in patients with BRAFV600E metastatic colorectal cancer (mCRC): efficacy and tumor markers. J Clin Oncol. (2018) 36(Suppl. 4):627 10.1200/JCO.2018.36.4_suppl.62729283749

[120] Sillo TO Beggs AD Morton DG Middleton G. Mechanisms of immunogenicity in colorectal cancer. Br J Surg. (2019) 106:1283–97. 10.1002/bjs.11204 31216061 PMC6772007

[121] Zhang Y Ertl HC. Starved and asphyxiated: how can CD8+ T cells within a tumor microenvironment prevent tumor progression. Front Immunol. (2016) 7:32. 10.3389/fimmu.2016.00032 26904023 PMC4748049

[122] Postow MA Callahan MK Wolchok JD Immune checkpoint blockade in cancer therapy. J Clin Oncol. (2015) 33:1974–82. 10.1200/JCO.2014.59.4358 25605845 PMC4980573

[123] Dierssen JWF de Miranda NF Ferrone S van Puijenbroek M Cornelisse CJ Fleuren GJ. HNPCC versus sporadic microsatellite-unstable colon cancers follow different routes toward loss of HLA class I expression. BMC Cancer. (2007) 7:33. 10.1186/1471-2407-7-33 17316446 PMC1808468

[124] Warabi M Kitagawa M Hirokawa K. Loss of MHC class II expression is associated with a decrease of tumor-infiltrating T cells and an increase of metastatic potential of colorectal cancer: immunohistological and histopathological analyses as compared with normal colonic mucosa and adenomas. Pathol Res Pract. (2000) 196:807–15. 10.1016/S0344-0338(00)80080-1 11156321

[125] Zhao Y Ge X He J Cheng Y Wang Z Wang J. The prognostic value of tumor-infiltrating lymphocytes in colorectal cancer differs by anatomical subsite: a systematic review and meta-analysis. World J Surg Oncol. (2019) 17:85. 10.1186/s12957-019-1621-9 31118034 PMC6532263

[126] Galon J Pagès F Marincola FM Thurin M Trinchieri G Fox BA. The immune score as a new possible approach for the classification of cancer. J Transl Med. 10:1. (2012) 10.1186/1479-5876-10-1 22214470 PMC3269368

[127] Galon J Mlecnik B Bindea G Angell HK Berger A Lagorce C. Towards the introduction of the ‘Immunoscore'in the classification of malignant tumours. J Pathol. (2014) 232:199–209. 10.1002/path.4287 24122236 PMC4255306

[128] Pagès F Mlecnik B Marliot F Bindea G Ou F-S Bifulco C. International validation of the consensus immunoscore for the classification of colon cancer: a prognostic and accuracy study. Lancet. (2018) 391:2128–39. 10.1016/S0140-6736(18)30789-X 29754777

[129] Li MX Liu XM Zhang XF Zhang JF Wang WL Zhu Y. Prognostic role of neutrophil-to-lymphocyte ratio in colorectal cancer: a systematic review and meta-analysis. Int J Cancer. (2014) 134:2403–13. 10.1002/ijc.28536 24122750

[130] Carruthers R Tho LM Brown J Kakumanu S McCartney E McDonald AC. Systemic inflammatory response is a predictor of outcome in patients undergoing preoperative chemoradiation for locally advanced rectal cancer. Colorectal Dis. (2012) 14:e701–7. 10.1111/j.1463-1318.2012.03147.x 22731833

[131] Climent M Ryan EJ Stakelum A Khaw YL Creavin B Lloyd A. Systemic inflammatory response predicts oncological outcomes in patients undergoing elective surgery for mismatch repair-deficient colorectal cancer. Int J Colorectal Dis. (2019) 34:1069–78. 10.1007/s00384-019-03274-6 30993458

[132] Minoo P Zlobec I Peterson M Terracciano L Lugli A. Characterization of rectal, proximal and distal colon cancers based on clinicopathological, molecular and protein profiles. Int J Oncol. (2010) 37:707–18. 10.3892/ijo_00000720 20664940

[133] Zhang L Zhao Y Dai Y Cheng J-N Gong Z Feng Y. Immune landscape of colorectal cancer tumor microenvironment from different primary tumor location. Front Immunol. (2018) 9:1578. 10.3389/fimmu.2018.01578 30042763 PMC6048410

[134] Arnold D Lueza B Douillard JY Peeters M Lenz HJ Venook A. Prognostic and predictive value of primary tumour side in patients with RAS wild-type metastatic colorectal cancer treated with chemotherapy and EGFR directed antibodies in six randomized trials. Ann Oncol. (2017) 28:1713–29. 10.1093/annonc/mdx175 28407110 PMC6246616

[135] Norris S Coleman A Kuri-Cervantes L Bower M Nelson M Goodier MR. PD-1 expression on natural killer cells and CD8^+^ T cells during chronic HIV-1 infection. Viral Immunol. (2012) 25:329–32. 10.1089/vim.2011.0096 22742708

[136] Bellucci R Martin A Bommarito D Wang K Hansen SH Freeman GJ. Interferon-gamma-induced activation of JAK1 and JAK2 suppresses tumor cell susceptibility to NK cells through upregulation of PD-L1 expression. Oncoimmunology. (2015) 4:e1008824. 10.1080/2162402X.2015.1008824 26155422 PMC4485824

[137] Llosa NJ Cruise M Tam A Wicks EC Hechenbleikner EM Taube JM. The vigorous immune microenvironment of microsatellite instable colon cancer is balanced by multiple counter-inhibitory checkpoints. Cancer Discov. (2015) 5:43–51. 10.1158/2159-8290.CD-14-0863 25358689 PMC4293246

[138] Holch JW Ricard I Stintzing S Modest DP Heinemann V. The relevance of primary tumour location in patients with metastatic colorectal cancer: a meta-analysis of first-line clinical trials. Eur J Cancer. (2017) 70:87–98. 10.1016/j.ejca.2016.10.007 27907852

[139] Chen YP Zhang J Wang YQ Liu N He QM Yang XJ. The immune molecular landscape of the B7 and TNFR immunoregulatory ligand-receptor families in head and neck cancer: a comprehensive overview and the immunotherapeutic implications. Oncoimmunology. (2017) 6:e1288329. 10.1080/2162402X.2017.1288329 28405520 PMC5384366

[140] Schmittnaegel M Rigamonti N Kadioglu E Cassara A Wyser Rmili C Kiialainen A. Dual angiopoietin-2 and VEGFA inhibition elicits antitumor immunity that is enhanced by PD-1 checkpoint blockade. Sci Transl Med. (2017) 9:eaak9670. 10.1126/scitranslmed.aak9670 28404865

[141] Lee Y Auh SL Wang Y Burnette B Wang Y Meng Y. Therapeutic effects of ablative radiation on local tumor require CD8+ T cells: changing strategies for cancer treatment. Blood. (2009) 114:589–95. 10.1182/blood-2009-02-206870 19349616 PMC2713472

[142] Twyman-Saint Victor C Rech AJ Maity A Rengan R Pauken KE Stelekati E. Radiation and dual checkpoint blockade activate non-redundant immune mechanisms in cancer. Nature. (2015) 520:373–7. 10.1038/nature14292 25754329 PMC4401634

[143] Anitei MG Zeitoun G Mlecnik B Marliot F Haicheur N Todosi AM. Prognostic and predictive values of the immunoscore in patients with rectal cancer. Clin Cancer Res. (2014) 20:1891–9. 10.1158/1078-0432.CCR-13-2830 24691640

[144] Teng F Mu D Meng X Kong L Zhu H Liu S. Tumor infiltrating lymphocytes (TILs) before and after neoadjuvant chemoradiotherapy and its clinical utility for rectal cancer. Am J Cancer Res. (2015) 5:2064–74. 26269765 PMC4529625

[145] Akiyoshi T Tanaka N Kiyotani K Gotoh O Yamamoto N Oba K. Immunogenomic profiles associated with response to neoadjuvant chemoradiotherapy in patients with rectal cancer. Br J Surg. (2019) 106:1381–92. 10.1002/bjs.11179 31197828

[146] Teng F Meng X Kong L Mu D Zhu H Liu S. Tumor-infiltrating lymphocytes, forkhead box P3, programmed death ligand-1, and cytotoxic T lymphocyte-associated antigen-4 expressions before and after neoadjuvant chemoradiation in rectal cancer. Transl Res. (2015) 166:721–32.e721. 10.1016/j.trsl.2015.06.019 26209749

[147] Bando H Tsukada Y Inamori K Fukuoka S Sasaki T Nishizawa Y P-228 VOLTAGE: multicenter phase Ib/II study of nivolumab monotherapy and subsequent radical surgery following preoperative chemoradiotherapy (CRT) with capecitabine in patients with locally advanced rectal cancer (LARC). Ann Oncol. (2018) 29(Suppl. 5):mdy151.227 10.1093/annonc/mdy151.227

